# Prediction of angiogenesis in extrahepatic cholangiocarcinoma using MRI-based machine learning

**DOI:** 10.3389/fonc.2023.1048311

**Published:** 2023-05-18

**Authors:** Jiong Liu, Mali Liu, Yaolin Gong, Song Su, Man Li, Jian Shu

**Affiliations:** ^1^Department of Radiology, The Affiliated Hospital of Southwest Medical University, Luzhou, Sichuan, China; ^2^Nuclear Medicine and Molecular Imaging Key Laboratory of Sichuan Province, Luzhou, Sichuan, China; ^3^Department of Hepatobiliary Surgery, The Affiliated Hospital of Southwest Medical University, Luzhou, China; ^4^Department of Research and Development, Shanghai United Imaging Intelligence Co., Shanghai, China

**Keywords:** cholangiocarcinoma, magnetic resonance imaging, machine learning, vascular endothelial growth factor, microvessel density

## Abstract

**Purpose:**

Reliable noninvasive method to preoperative prediction of extrahepatic cholangiocarcinoma (eCCA) angiogenesis are needed. This study aims to develop and validate machine learning models based on magnetic resonance imaging (MRI) for predicting vascular endothelial growth factor (VEGF) expression and the microvessel density (MVD) of eCCA.

**Materials and methods:**

In this retrospective study from August 2011 to May 2020, eCCA patients with pathological confirmation were selected. Features were extracted from T1-weighted, T2-weighted, and diffusion-weighted images using the MaZda software. After reliability testing and feature screening, retained features were used to establish classification models for predicting VEGF expression and regression models for predicting MVD. The performance of both models was evaluated respectively using area under the curve (AUC) and Adjusted R-Squared (Adjusted R^2^).

**Results:**

The machine learning models were developed in 100 patients. A total of 900 features were extracted and 77 features with intraclass correlation coefficient (ICC) < 0.75 were eliminated. Among all the combinations of data preprocessing methods and classification algorithms, Z-score standardization + logistic regression exhibited excellent ability both in the training cohort (average AUC = 0.912) and the testing cohort (average AUC = 0.884). For regression model, Z-score standardization + stochastic gradient descent-based linear regression performed well in the training cohort (average Adjusted R^2 = ^0.975), and was also better than the mean model in the test cohort (average Adjusted R^2 = ^0.781).

**Conclusion:**

Two machine learning models based on MRI can accurately predict VEGF expression and the MVD of eCCA respectively.

## 1 Introduction

Cholangiocarcinoma (CCA) is a group of highly heterogeneous malignancies. CCA can be divided into three subtypes: intrahepatic cholangiocarcinoma (iCCA), perihilar cholangiocarcinoma (pCCA) and distal cholangiocarcinoma (dCCA). pCCA and dCCA are collectively referred to as extrahepatic cholangiocarcinoma (eCCA), accounting for 80–90% of all types of CCA (1). Improvements in diagnosis and treatment have stabilized or decreased the morbidity and mortality of eCCA in most areas (1, 2). Although surgery has played an essential role, more oncologists have emphasized the necessity for neoadjuvant therapy, including vascular‐targeted therapy.

CCA is traditionally regarded as a lymphovascular tumor with a rich polymorphic tumor microenvironment, and the overexpression of microvessels has a strong correlation with tumors (3). Vascular‐targeted therapy mainly inhibits tumor-associated angiogenesis through drugs (e.g. bevacizumab). Angiogenesis is an important factor for maintain the rapid growth and metastasis of malignant tumors, providing necessary oxygen and nutrients to tumor cells (4, 5). Vascular endothelial growth factor (VEGF), a kind of homodimeric heparin-binding protein, can enhance the division capability of vascular endothelial cells and promote tumor-associated angiogenesis (6). Poor T cell infiltration and high M2-TAM in eCCA are correlated with elevated VEGF levels (7). In addition, the 5-year survival rate of eCCA patients with high microvessel density MVD (2.2%) was significantly worse than low MVD patients (42.1%) (8, 9). It is undeniable that VEGF and MVD are indeed related to the prognosis and progression of almost all tumors, and this is also true in eCCA (10), and about 59% of eCCA patients overexpress VEGF (11). Currently, immunohistochemical stains and microarray analysis are most commonly used to detect VEGF expression and MVD. However, this method is invasive and difficult to repeat.

Magnetic resonance imaging (MRI) can clearly visualize various biliary diseases (12). However, naked-eye evaluation of the tumor VEGF level and MVD still remains extremely challenging. Machine learning, which can deeply mine images and analyze them objectively and quantitatively, has become a commonly used method in clinical oncology research (13–15). One study showed that six pathological features of iCCA, including VEGF, can be evaluated accurately by machine learning of ultrasound images before operation. The area under the curve (AUC) for the VEGF group was 0.86 (16). However, carcinogenesis, diagnosis, and treatment markedly differ between iCCA and eCCA (1, 17). Additionally, the ultrasound has great variability according to the level of the operators. Additionally, MVD belongs to continuous numerical data, and there is no exact cut-off value. Thus, most related studies use the mean or median value of MVD as cut-off (1, 8). However, this method is controversial for the clinical interpretation of different patient samples. In this study, we constructed a classification model for prediction of VEGF expression and a regression model for quantitative prediction of MVD based on multi-sequence MR images, using machine learning for objective and non-invasive preoperative evaluation of VEGF expression and MVD of eCCA.

## 2 Materials and methods

### 2.1 Patients’ enrollment

**Figure 1:**
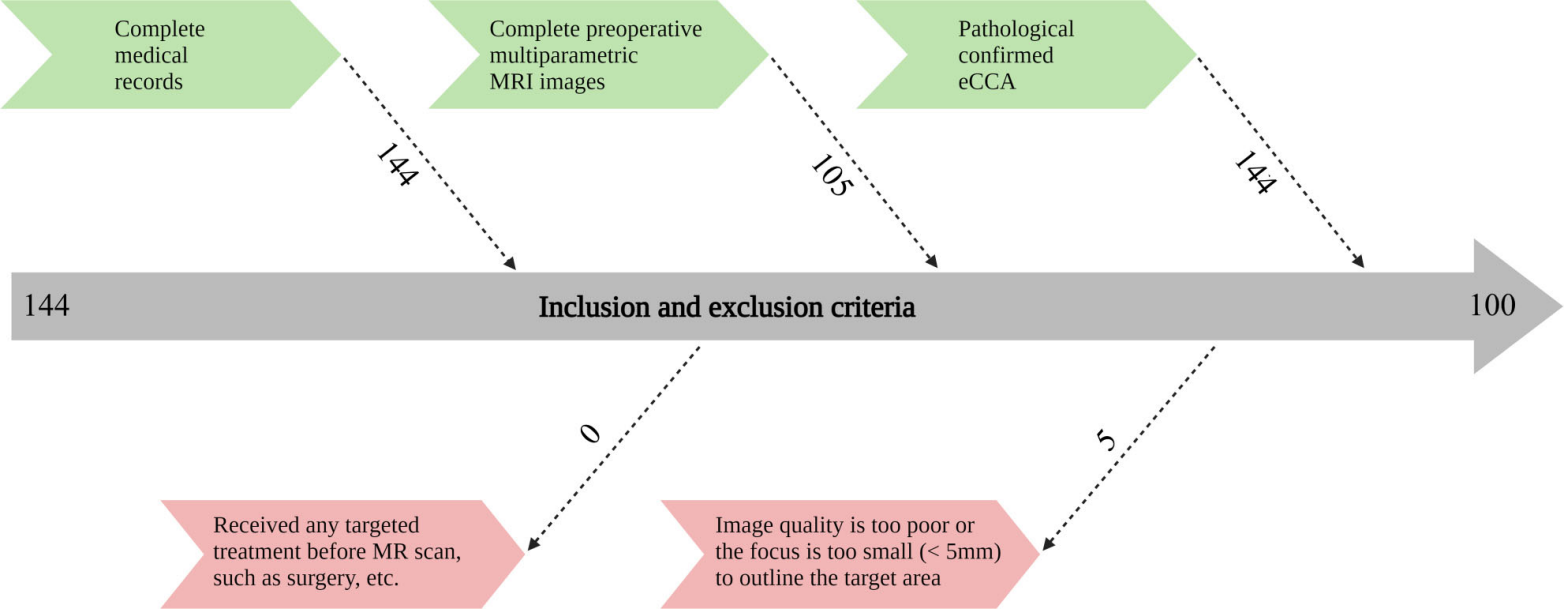
Flow chart of inclusion and exclusion criteria of patients. MRI, magnetic resonance imaging; eCCA, extrahepatic cholangiocarcinoma.

This retrospective study was approved by the institutional review board, and the human-related procedures followed the “Helsinki Declaration”. Since the study was retrospective, written informed consent of patients was not required. We collected patients treated in our hospital from January 2011 to December 2020 and met the following inclusion and exclusion criteria. Inclusion criteria included: (I) had complete medical records; (II) had complete preoperative multiparametric MR images; (III) pathologically confirmed eCCA. Exclusion criteria included: (I) the patient had received any treatment before MR scan, such as surgery, and targeted treatment; (II) the image quality was too poor or the focus was too small (< 5 mm) to outline the focus target area. The flow diagram of patient enrollment is displayed in **Figure 1**.

### 2.2 Pathological specimen processing

All enrolled patients underwent surgical resection. Tumor specimens obtained during surgery were used for pathological analysis to determine VEGF expression and MVD count. Immunohistochemical staining and microarray analysis of VEGF and MVD were performed according to relevant standard methods by a pathologist with more than 10 years of clinical experience.

### 2.3 MR image acquisition

Preoperative evaluation included standard upper abdominal scanning with 3.0T MRI scanner (Achieva, Philips, Netherlands) and 16-channel trunk coil. MRI acquisition sequence included but was not limited to transverse T1-weighted imaging (T1WI), T2-weighted imaging (T2WI), and diffusion-weighted imaging (DWI). The parameters of these three acquisition sequences are detailed in **Table 1**. All MR images were retrieved and analyzed by the Picture Archiving and Communication Systems. In addition, all MR images applied voxel size normalization and voxel intensity normalization.

**Table 1:** MRI sequences and parameters.

Parameter	T1WI	T2WI	DWI
TR (msec)	3.1	1610	934
TE (msec)	1.44	70	52
Section thickness (mm)	3	7	7
Section gap (mm)	1.5	1	1
FOV (mm^2^)	280 × 305	280 × 305	280 × 305
Matrix size	244 × 186	176 × 201	100 × 124
Flip angle (°)	10	90	90
b values (s/mm2)	–	–	0 and 800

MRI, magnetic resonance imaging; T1WI, T1-weighted imaging; T2WI, T2-weighted imaging; DWI, diffusion-weighted imaging; TR, repetition time; TE, echo time; FOV, field of view.

### 2.4 Image segmentation and features extraction

Region of interest (ROI) segmentation and feature extraction were performed by an experienced radiologist using the software MaZda (version 4.6, http://www.eletel.p.lodz.pl/programy/mazda/) (18–20). The ROI margins were strictly defined to always be 1–2 mm from the tumor margin. In addition, the “± 3 sigma” option in the MaZda software was selected for image standardization.

### 2.5 Intra-observer and inter-observer agreement

To assess the stability of features, two radiologists jointly selected T1WI, T2WI, and DWI images of 20 patients at random for repeated segmentation. One radiologist re-outlined the ROI twice at different times of the week. Another radiologist independently outlined the ROI once. The extracted features were used for ICC calculation using Python programming language (version 3.7, https://www.python.org) to evaluate the intra-observer and inter-observer agreement of each feature. ICC > 0.75 indicated good reliability, and this feature was retained.

### 2.6 Feature processing and model building

**Figure 2:**
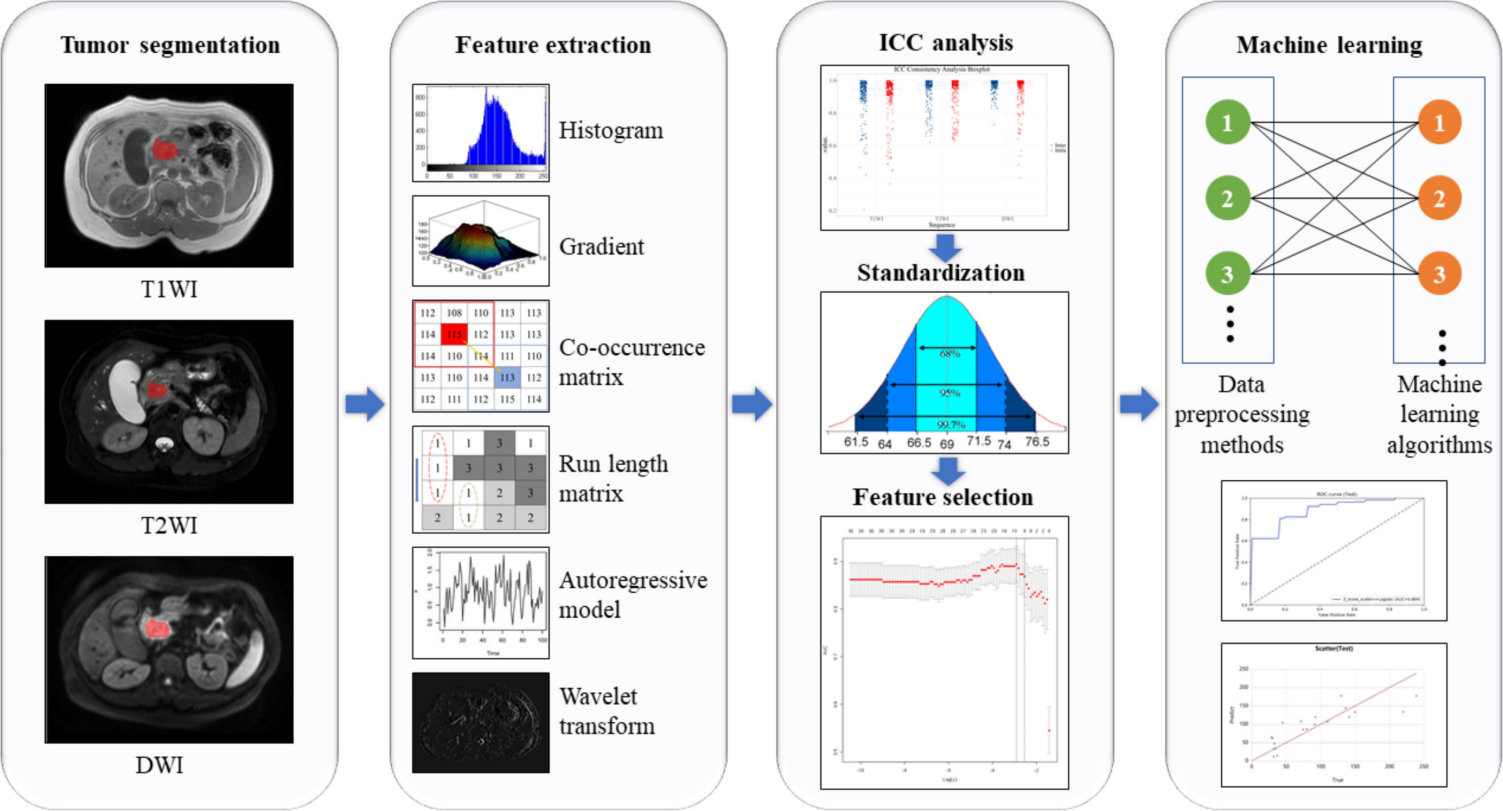
Workflow of machine learning process in the current study.

Feature processing and model building were performed with the uAI Research Portal (United Imaging Intelligence, China). First, 80% of the samples were randomly selected as the training cohort and the other 20% as the test cohort. Then, Z-score standardization was used to eliminate errors caused by different dimensions. The least absolute shrinkage and selection operator (Lasso) regression method was used for feature selection. When constructing both models, eight data preprocessing methods were tried: Box-cox transform, L1-norm regularization, L2-norm regularization, max abs normalization, min-max normalization, Quantile transform, YeoJohnson transform, and Z-score standardization. Finally, when constructing the classification model for predicting VEGF expression, nine machine learning algorithms were tried: Gaussian process regression, K-nearest neighbors, logistic regression, partial least squares-discriminant analysis, quadratic discriminant analysis, random forest, stochastic gradient descent based linear regression, support vector machine, and XGboost. In addition to logistic regression, partial least squares-discriminant analysis, quadratic discriminant analysis, the other six algorithms were also used to construct the regression model for predicting MVD. The above steps were repeated 20 times to ensure good reliability of the models. An overview of the machine learning workflow is shown in **Figure 2**.

AUC of the subject ROC and the Adjusted R-Squared (Adjusted R^2^) were used to evaluate the effectiveness of the classification model and the regression model, respectively. Other auxiliary evaluation indices including F1 score, recall, precision, sensitivity, specificity, accuracy, mean square error (MSE), mean absolute error (MAE), and Pearson correlation coefficient (PCC) were also calculated. Finally, the bias and variance of both models were calculated to evaluate their fitting and generalization. The models with the highest average AUC or Adjusted R^2^ in the test cohort were identified as the best models for classification or regression.

### 2.7 Statistical analysis

Statistical analysis of the data on clinical and pathological characteristics of patients was performed using Statistical Product and Service Solutions (SPSS, version 25.0, IBM). Continuous variables were expressed as mean ± standard deviation (SD) when they followed a normal distribution, and median value was used for non-normally distributed data. The correlation between VEGF expression and age, gender, tumor location, and MVD was evaluated using binary multivariate logistic regression. The evaluation indices of both machine learning models were calculated using the uAI Research Portal. All statistical tests were two-sided, and *P* values < 0.05 were considered significant.

## 3 Results

### 3.1 Patient characteristics

There are 105 patients were in accordance with the inclusive criteria. No patients were excluded as the reason that they received any treatment before MRI. However, 5 patients were excluded because the image quality was too poor or the lesion was too small. Finally, we identified 100 eligible patients based on the inclusion and exclusion criteria. The mean age of all eCCA patients was 57.38 years. The ratio of male to female and pCCA patients to dCCA patients was close to 50%. Additionally, there were more patients with positive VEGF expression than negative. The detailed results are shown in **Table 2**.

**Table 2:** Clinical and histologic characteristics of all eCCA patients.

Variable	Whole (*n* = 100)
Age, mean ± SD, years	57.38 ± 10.06
Gender	
Female, *n* Male, *n*	46 54
Localization	
pCCA, *n* dCCA, *n*	47 53
MVD, mean ± SD	101.16 ± 58.11
VEGF	
Positive, *n* Negative, *n*	71 29

SD, standard deviation; pCCA, perihilar cholangiocarcinoma; dCCA, distal cholangiocarcinoma; VEGF, vascular endothelial growth factor; MVD, microvessel density.

*, *P* < 0.05.

In addition, in the multivariable logistic regression analysis of the related factors of VEGF expression, age (*P* = 0.125, OR = 0.461), gender (*P* = 0.059, OR = 0.952), and tumor (*P* = 0.583, OR = 0.764) location did not affect the expression of VEGF, while there was a significant positive relationship (*P* = 0.008, OR = 1.014) between MVD and VEGF expression. Likelihood ratio test (χ² (4) = 16.670, *P* = 0.002) and Hosmer–Lemeshow test (χ² (8) = 13.278, p = 0.103) showed the validity and goodness of fit of the multivariable logistical regression analysis model.

### 3.2 Features extraction

More than 300 image features were extracted from each ROI using MaZda. Then, the features with missing values were deleted. Finally, each sequence image uniformly retained 300 features. These features were classified into six feature families including histogram (12), gradient (6), co-occurrence matrix (20), run length matrix (240), autoregressive model (6), and wavelet (16). Finally, the features extracted from T1WI, T2WI, and DWI images of each patient were mixed, and a total of 900 features were obtained.

### 3.3 Intra-observer and inter-observer agreement

**Figure 3:**
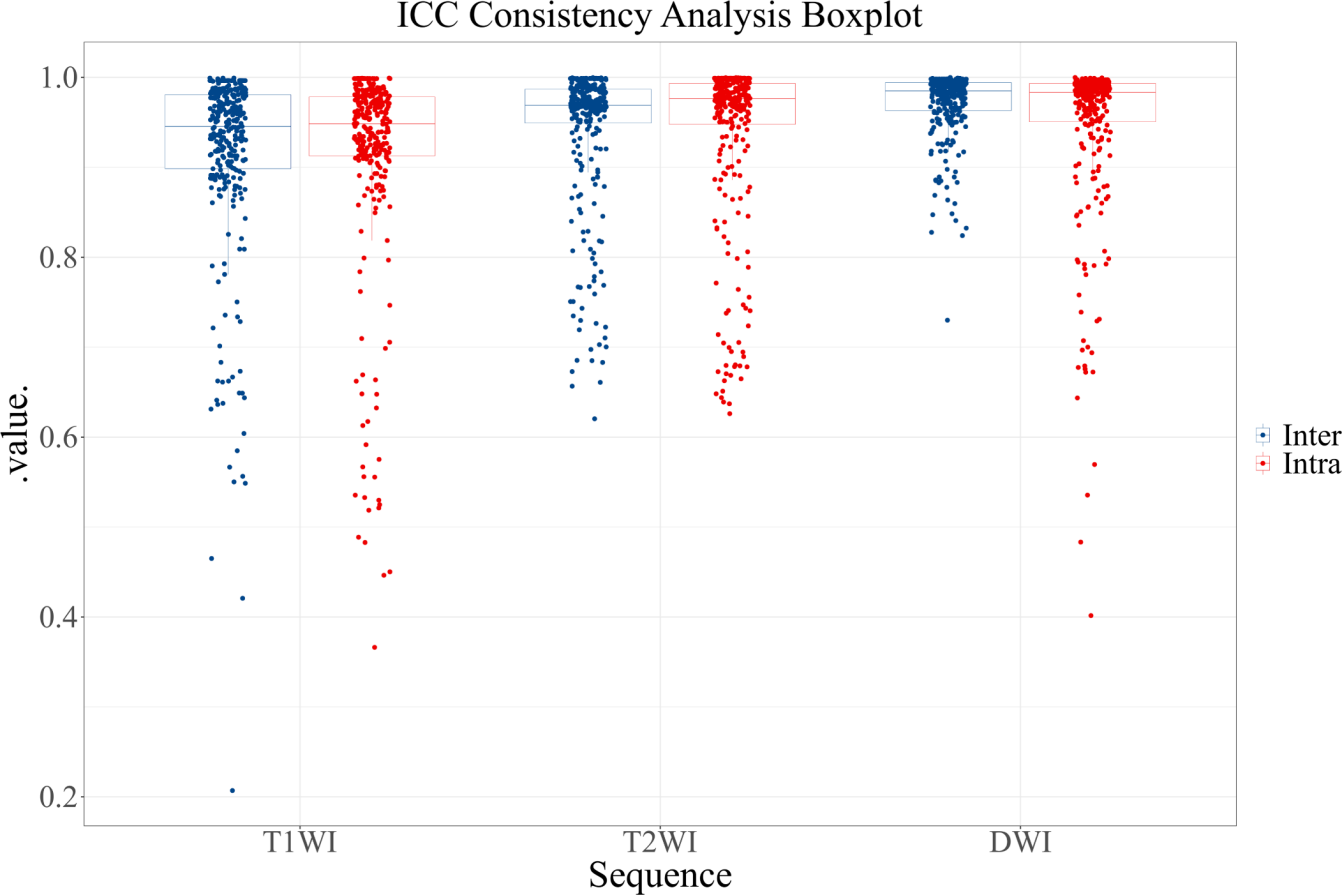
ICC consistency analysis boxplot. Blue represents inter-observer agreement, and red represents intra-observer agreement.

Through ICC consistency analysis, 823 features with both intra- and inter-observer ICC values greater than 0.75 were identified among the 900 features. The removed features included 30 T1WI image features, 29 T2WI image features, and 18 DWI image features. **Figure 3** shows the results of the ICC consistency analysis for each feature.

### 3.4 Feature selection and models construction

**Figure 4:**
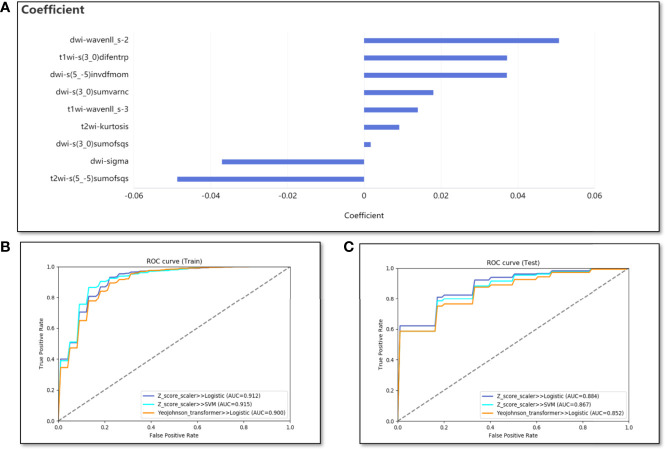
The performance of the classification model. **(A)** The bar graph shows the weight coefficient of each predictive feature in the model of Z-score standardization + logistic regression. **(B, C)** The ROC curves for training and test cohorts of different combinations (Three combinations with the best results are listed).

In the classification model for predicting VEGF expression, the nine best features were obtained by selection using Lasso with an alpha value of 0.075. Five of them were DWI image features, namely DWI-sigma, DWI-s(3_0)sumofsqs, DWI-s(3_0)sumvarnc, DWI-s(5_-5)invdfmom, and DWI-wavenll_s-2. The other four features were T1WI-s(3_0)difentrp, T1WI-wavenll_s-3, T2WI-s(5_-5) sumofsqs, and T2WI-kurtosis (**Figure 4A**). Based on the nine features, 72 different combinations of machine learning classification models were constructed. Finally, the combination with the highest average AUC value in the test cohort was the Z-score standardization + logistic regression. The average AUCs of the training and test cohorts were 0.912 (range, 0.876–0.963) and 0.884 (range, 0.631–1), respectively (**Figures 4B, C**). The average accuracy and sensitivity of the model in the test cohort were also excellent, 0.84 (range, 0.65–0.952) and 0.926 (range, 0.786–1), respectively. The average specificity in the test cohort was relatively poor, at only 0.633 (range, 0.333–0.833).

**Figure 5:**
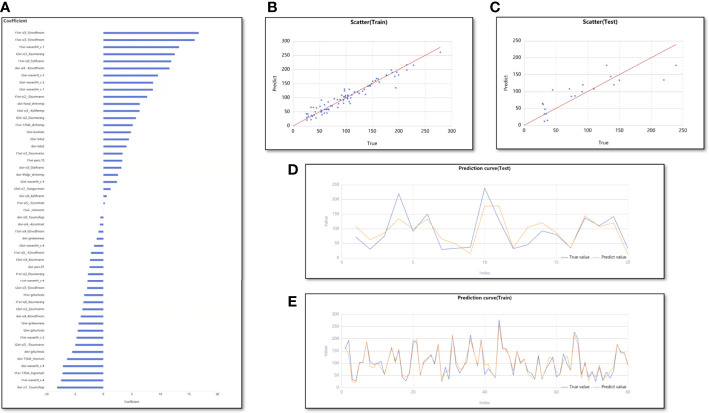
The performance of the Z-score standardization + stochastic gradient descent based linear regression. **(A)** The bar graph shows the weight coefficient of top 50 predictive feature. **(B, C)** The scatter plots for training and test cohorts. **(D, E)** The prediction curves for training and test cohorts.

In the regression model for predicting MVD, 66 features were retained using Lasso with an alpha value of 1.000. Of these, the number of T1WI, T2WI, and DWI image features were 22, 25, and 19, respectively (**Figure 5A**). Using these 66 features, 48 machine learning regression models were constructed. Lastly, the model of the Z-score standardization + stochastic gradient descent based linear regression showed good performance and was chosen as the best model. The average Adjusted R^2^ of its training and test cohorts were 0.975 (range, 0.964–0.984) and 0.781 (range, 0.233–0.896), respectively. The results of the average Adjusted R^2^ in both the training and test cohorts were acceptable, and their values were greater than the mean model. The scatter plots (**Figures 5B, C**) and prediction curves (**Figures 5D, E**) display the prediction results and trends for each sample. **Table 3** shows the results of all evaluation indices for the two models predicting VEGF expression and MVD.

**Table 3:** Performance evaluation of two models for predicting VEGF expression and MVD.

Evaluation metrics	Classification model		Regression model	
	Training cohorts (80 patients)	Testing cohorts (20 patients)	Training cohorts (80 patients)	Testing cohorts (20 patients)
AUC	0.912	0.884		
F1-score	0.923	0.891		
Precision	0.887	0.864		
Sensitivity	0.961	0.926		
Specificity	0.701	0.633		
Accuracy	0.886	0.84		
R^2^			0.927	0.434
MAE			11.409	34.374
MSE			245	1891.407
PCC			0.963	0.725
Bias	0.129		2327.009	
Variance	0.062		2902.103	

VEGF, vascular endothelial growth factor; MVD, microvessel density; AUC, area under the curve; R^2^, coefficient of determination; MAE, mean absolute error; MSE, mean square error; PCC, Pearson correlation coefficient.

## 4 Discussion

In this study, we established two machine learning models based on MR images to predict VEGF expression and MVD in eCCA. When constructing the machine learning model for predicting MVD, we used the regression model, which is rarely used in medical research machine learning, and obtained satisfactory results. The classification model successfully predicted the expression of VEGF in eCCA. The regression model for predicting MVD also exhibited excellent performance. This demonstrates that machine learning is promising for the clinical evaluation of tumor-associated angiogenesis in eCCA.

Recent studies have shown that VEGF overexpression and MVD are related to tumor progression, metastasis, and prognosis in eCCA (8, 9, 21). For unresectable middle and advanced eCCA patients, the effect of conventional chemotherapy is not satisfactory (22, 23). Therefore, researchers are exploring new treatment protocols for the molecular pathways (such as tumor-associated angiogenesis) in the occurrence and development of CCA. Currently, the application of purely vascular targeted therapies in CCA patients is limited. However, studies have shown that combining vascular targeted therapies with immunotherapies will bring significant benefits to patients (24–26). In addition, in clinical practice, invasive histopathological examinations for monitoring angiogenesis within tumors still presents many problems and inconvenience. Some scholars have used conventional imaging methods to study the angiogenesis of CCA. Park et al. retrospectively analyzed the CT images of 147 patients with iCCA. They found that high blood supply on CT images was related to higher relapse-free survival and better prognosis, and the vascular distribution on CT images could be used as a non-invasive prognostic index for iCCA (27). Furthermore, Yugawa et al. confirmed that MVD in CCA tumors was closely related to radiological characteristics of the hepatic arterial phase on enhanced CT, which may be a potential prognostic indicator (28).

In the research studied to date, the number of molecular or pathological studies of CCA using machine learning is still relatively rare, and most of the research subjects are iCCA. Studies by Sadot et al. indicated that quantitative imaging phenotypes in CT images correlated with the expression of specific hypoxic markers in iCCA, including VEGF (29). Recently, Zhou and his team established a machine learning model based on dynamic contrast-enhanced MR images whose features can be used to preoperatively predict microvascular invasion in patients with mass-forming iCCA (30). Prior studies have shown, the pathological features of eCCA (including pathological grading, lymph node metastasis, T stage, perineural infiltration, and microvascular infiltration) can also be predicted by several machine learning models with excellent results (31, 32).

In the present work, our machine learning models, based on MR images and used to predict the VEGF expression and MVD of eCCA, further enriched the knowledge in the field of eCCA machine learning and provides credible aid for the treatment and prognosis of patients with eCCA. In addition, our exploration of regression-based machine learning for continuous variables in which optimal cut-off values are not clinically available was effective. More interestingly, we found that the DWI image features accounted for a relatively large number (5/9) of the classification model features, while the number of T2WI image features was larger (25/66) in the regression model. The reasons for this result may be varied, such as correlation between different types of images and predicted objects or systematic error caused by inconsistent algorithms in the process of machine learning. In addition, in both the classification and regression models, the feature with the largest weight coefficient was the wavelet transform feature in the DWI image. This is in agreement with the previous research results of several other scholars (30, 33, 34), indicating that wavelet transform features may be able to characterize tumor biology on multiple scales.

There are scenarios in which the present studies fall short. First, this is a retrospective case-control study with a small sample size and all patients were from a single institution, so selection bias may be present. To increase the universality of the model application, it is necessary to carry out prospective multicenter studies with larger samples. Second, this study only included conventional non-enhanced MRI sequences and did not include enhancement sequences and other special sequences. On the one hand, almost all patients underwent conventional sequence scans, which is conducive to increasing the applicability of the model. On the other hand, adding sequence types would further reduce the number of patient samples included in the study. Last but not least, radiomics mines the deep feature information hidden in the image, which can not be recognized by the naked eye, and may be related to the disease itself, regardless of whether it is enhanced or not. Some studies have shown that enhanced images may not significantly improve the efficiency of radiomics models compared with non-enhanced images (35). Third, tumors smaller than 5 mm were excluded during this study, making it difficult for the machine learning models to predict tumors of smaller size. Finally, the number of features in the regression model was relatively large. In future work, we will test further methods and algorithms to obtain the minimum number of features and the best model efficiency.

In this study, we constructed and internally validated MRI-based machine learning models to predict VEGF expression and MVD in eCCA. Both models provide powerful guidance for monitoring eCCA angiogenesis, may assist in clinical decision-making, and ultimately improve the prognosis of patients.

## Data availability statement

The raw data supporting the conclusions of this article will be made available by the authors, without undue reservation.

## Author contributions

JS and JL contributed to conception and design. JL, MLL and YLG contributed to the collection and arrangement of data. JL and ML contributed to data analysis and manuscript writing. JS and SS edited the manuscript. All authors contributed to the article and approved the submitted version.

## References

[1] Banales JM Marin JJG Lamarca A Rodrigues PM Khan SA Roberts LR. Cholangiocarcinoma 2020: the next horizon in mechanisms and management. Nat Rev Gastroenterol Hepatol (2020) 17(9):557–88. doi: 10.1038/s41575-020-0310-z PMC7447603 32606456

[2] Bertuccio P Malvezzi M Carioli G Hashim D Boffetta P El-Serag HB. Global trends in mortality from intrahepatic and extrahepatic cholangiocarcinoma. J Hepatol (2019) 71(1):104–14. doi: 10.1016/j.jhep.2019.03.013 30910538

[3] Mariotti V Fiorotto R Cadamuro M Fabris L Strazzabosco M. New insights on the role of vascular endothelial growth factor in biliary pathophysiology. JHEP Rep (2021) 3(3):100251. doi: 10.1016/j.jhepr.2021.100251 34151244 PMC8189933

[4] Jayson GC Kerbel R Ellis LM Harris AL. Antiangiogenic therapy in oncology: current status and future directions. Lancet (2016) 388(10043):518–29. doi: 10.1016/S0140-6736(15)01088-0 26853587

[5] Butler JM Kobayashi H Rafii S. Instructive role of the vascular niche in promoting tumour growth and tissue repair by angiocrine factors. Nat Rev Cancer (2010) 10(2):138–46. doi: 10.1038/nrc2791 PMC2944775 20094048

[6] Ellis LM Hicklin DJ. VEGF-targeted therapy: mechanisms of anti-tumour activity. Nat Rev Cancer (2008) 8(8):579–91. doi: 10.1038/nrc2403 18596824

[7] King G Javle M. FGFR inhibitors: clinical activity and development in the treatment of cholangiocarcinoma. Curr Oncol Rep (2021) 23(9):108. doi: 10.1007/s11912-021-01100-3 34269915

[8] Möbius C Demuth C Aigner T Wiedmann M Wittekind C Mössner J. Evaluation of VEGF a expression and microvascular density as prognostic factors in extrahepatic cholangiocarcinoma. Eur J Surg Oncol (2007) 33(8):1025–9. doi: 10.1016/j.ejso.2007.02.020 17400419

[9] Thelen A Scholz A Benckert C Schröder M Weichert W Wiedenmann B. Microvessel density correlates with lymph node metastases and prognosis in hilar cholangiocarcinoma. J Gastroenterol (2008) 43(12):959–66. doi: 10.1007/s00535-008-2255-9 19107340

[10] Calastri MCJ Ferreira RF Tenani GD Spinola LP Vieira GF Rabaça Roque Botelho MF. Investigating VEGF. miR-145-3p, and miR-101-3p expression in patients with cholangiocarcinoma. Asian Pac J Cancer Prev (2022) 23(7):2233–41. doi: 10.31557/APJCP.2022.23.7.2233 PMC9727337 35901327

[11] Wang M Chen Z Guo P Wang Y Chen G. Therapy for advanced cholangiocarcinoma: current knowledge and future potential. J Cell Mol Med (2021) 25(2):618–28. doi: 10.1111/jcmm.16151 PMC7812297 33277810

[12] Ringe KI Wacker F. Radiological diagnosis in cholangiocarcinoma: application of computed tomography, magnetic resonance imaging, and positron emission tomography. Best Pract Res Clin Gastroenterol (2015) 29(2):253–65. doi: 10.1016/j.bpg.2015.02.004 25966426

[13] Liu Z Wang S Dong D Wei J Fang C Zhou X. The applications of radiomics in precision diagnosis and treatment of oncology: opportunities and challenges. Theranostics (2019) 9(5):1303–22. doi: 10.7150/thno.30309 PMC6401507 30867832

[14] Lambin P Leijenaar RTH Deist TM Peerlings J de Jong EEC van Timmeren J. Radiomics: the bridge between medical imaging and personalized medicine. Nat Rev Clin Oncol (2017) 14(12):749–62. doi: 10.1038/nrclinonc.2017.141 28975929

[15] Tsilimigras DI Mehta R Moris D Sahara K Bagante F Paredes AZ. A machine-based approach to preoperatively identify patients with the most and least benefit associated with resection for intrahepatic cholangiocarcinoma: an international multi-institutional analysis of 1146 patients. Ann Surg Oncol (2020) 27(4):1110–9. doi: 10.1245/s10434-019-08067-3 31728792

[16] Peng YT Zhou CY Lin P Wen DY Wang XD Zhong XZ. Preoperative ultrasound radiomics signatures for noninvasive evaluation of biological characteristics of intrahepatic cholangiocarcinoma. Acad Radiol (2020) 27(6):785–97. doi: 10.1016/j.acra.2019.07.029 31494003

[17] Rizvi S Gores GJ. Pathogenesis, diagnosis, and management of cholangiocarcinoma. Gastroenterology (2013) 145(6):1215–29. doi: 10.1053/j.gastro.2013.10.013 PMC3862291 24140396

[18] Strzelecki M Szczypinski P Materka A Klepaczko A. A software tool for automatic classification and segmentation of 2D/3D medical images. Nucl Instruments Methods Phys Res Section A: Accelerators Spectrometers Detectors Associated Equipment (2013) 702:137–40. doi: 10.1016/j.nima.2012.09.006

[19] Szczypiński PM Strzelecki M Materka A Klepaczko A. MaZda–a software package for image texture analysis. Comput Methods Programs Biomed (2009) 94(1):66–76. doi: 10.1016/j.cmpb.2008.08.005 18922598

[20] Szczypinski PM Strzelecki M Materka A. Mazda-A software for texture analysis, in: 2007 international symposium on information technology convergence (ISITC 2007). (2007) 245–9.

[21] Xu D Li J Jiang F Cai K Ren G. The effect and mechanism of vascular endothelial growth factor (VEGF) on tumor angiogenesis in gallbladder carcinoma. Iran J Public Health (2019) 48(4):713–21. PMC6500531 31110982

[22] Neuzillet C Casadei-Gardini A Brieau B Vivaldi C Brandi G Tougeron D. Fluropyrimidine single agent or doublet chemotherapy as second line treatment in advanced biliary tract cancer. Int J Cancer (2020) 147(11):3177–88. doi: 10.1002/ijc.33146 32525595

[23] Fornaro L Vivaldi C Cereda S Leone F Aprile G Lonardi S. Second-line chemotherapy in advanced biliary cancer progressed to first-line platinum-gemcitabine combination: a multicenter survey and pooled analysis with published data. J Exp Clin Cancer Res (2015) 34:156. doi: 10.1186/s13046-015-0267-x 26693938 PMC4689003

[24] Ma Z Li H Liu L. Combining PD-1 inhibitor with VEGF/VEGFR2 inhibitor in chemotherapy: report of a patient with end-stage cholangiocarcinoma and review of literature. Recent Pat Anticancer Drug Discov (2021) 16(1):101–7. doi: 10.2174/1574892815999201231215311 33390149

[25] Hack SP Verret W Mulla S Liu B Wang Y Macarulla T. IMbrave 151: a randomized phase II trial of atezolizumab combined with bevacizumab and chemotherapy in patients with advanced biliary tract cancer. Ther Adv Med Oncol (2021) 13:17588359211036544. doi: 10.1177/17588359211036544 34377158 PMC8326820

[26] Yuan M Zhu Z Mao W Wang H Qian H Wu J. Anlotinib combined with anti-PD-1 antibodies therapy in patients with advanced refractory solid tumors: a single-center, observational, prospective study. Front Oncol (2021) 11:683502. doi: 10.3389/fonc.2021.683502 34692475 PMC8529018

[27] Park HM Jang HY Lee DE Kang MJ Han SS Kim SW. Prognostic impact of tumor vascularity on CT in resectable intrahepatic cholangiocarcinoma. HPB (Oxford) (2022) 24(3):359–69. doi: 10.1016/j.hpb.2021.06.424 34325966

[28] Yugawa K Itoh S Yoshizumi T Iseda N Tomiyama T Toshima T. Prognostic impact of tumor microvessels in intrahepatic cholangiocarcinoma: association with tumor-infiltrating lymphocytes. Mod Pathol (2021) 34(4):798–807. doi: 10.1038/s41379-020-00702-9 33077921

[29] Sadot E Simpson AL Do RK Gonen M Shia J Allen PJ. Cholangiocarcinoma: correlation between molecular profiling and imaging phenotypes. PloS One (2015) 10(7):e0132953. doi: 10.1371/journal.pone.0132953 26207380 PMC4514866

[30] Zhou Y Zhou G Zhang J Xu C Wang X Xu P. Radiomics signature on dynamic contrast-enhanced MR images: a potential imaging biomarker for prediction of microvascular invasion in mass-forming intrahepatic cholangiocarcinoma. Eur Radiol (2021) 31(9):6846–55. doi: 10.1007/s00330-021-07793-1 33638019

[31] Yang C Huang M Li S Chen J Yang Y Qin N. Radiomics model of magnetic resonance imaging for predicting pathological grading and lymph node metastases of extrahepatic cholangiocarcinoma. Cancer Lett (2020) 470:1–7. doi: 10.1016/j.canlet.2019.11.036 31809800

[32] Huang X Shu J Yan Y Chen X Yang C Zhou T. Feasibility of magnetic resonance imaging-based radiomics features for preoperative prediction of extrahepatic cholangiocarcinoma stage. Eur J Cancer (2021) 155:227–35. doi: 10.1016/j.ejca.2021.06.053 34391055

[33] Xu X Zhang HL Liu QP Sun SW Zhang J Zhu FP. Radiomic analysis of contrast-enhanced CT predicts microvascular invasion and outcome in hepatocellular carcinoma. J Hepatol (2019) 70(6):1133–44. doi: 10.1016/j.jhep.2019.02.023 30876945

[34] Liang W Xu L Yang P Zhang L Wan D Huang Q. Novel nomogram for preoperative prediction of early recurrence in intrahepatic cholangiocarcinoma. Front Oncol (2018) 8:360. doi: 10.3389/fonc.2018.00360 PMC6131601 30234019

[35] Dong J Li S Li L Liang S Zhang B Meng Y. Differentiation of paediatric posterior fossa tumours by the multiregional and multiparametric MRI radiomics approach: a study on the selection of optimal multiple sequences and multiregions. Br J Radiol (2022) 95(1129):20201302. doi: 10.1259/bjr.20201302 34767476 PMC8722235

